# Novel *CFTR* Mutations in Two Iranian Families with Severe Cystic Fibrosis

**DOI:** 10.7508/ibj.2016.04.003

**Published:** 2016

**Authors:** Marzieh Mohseni, Mohammad Razzaghmanesh, Elham Parsi Mehr, Hanieh Zare, Maryam Beheshtian, Hossein Najmabadi

**Affiliations:** 1 Kariminejad-Najmabadi Pathology and Genetics Center, Tehran, Iran; 2 Genetics Research Center, University of Social Welfare and Rehabilitation Sciences, Tehran, Iran

**Keywords:** Cystic fibrosis, Cystic fibrosis transmembrane conductance regulator protein, Mutation, Sequence analysis, Iran

## Abstract

**Background:**

Cystic fibrosis (CF) is a common autosomal recessive disorder that affects many body systems and is produced by mutations in the cystic fibrosis transmembrane conductance regulator (*CFTR*) gene. CF is also the most frequently inherited disorder in the West. The aim of this study was to detect the mutations in the *CFTR* gene in two Iranian families with CF.

**Methods:**

After DNA extraction using the salting out method, a mutation panel consisting of 35 common mutations was tested by PCR, followed by reverse hybridization Strip Assay. To confirm the mutations, we have also performed Sanger sequencing for all 27 exons, intronic flanking regions, and 5’ and 3’ UTRs of the *CFTR* gene.

**Results:**

Carrier testing in a spouse revealed a novel nonsense mutation in the *CFTR* gene (c.2777 T>A (p.L926X)) in exon 17 for husband and a previously described heterozygous splice site pathogenic mutation (c.1393-1G>A) in his wife. The other novel compound heterozygous missense mutation (c.3119 T>A (p.L1040H)), which was previously reported as nonsense c.3484C>T (p.R1162X) mutation, was found in exon 19 in patient screening.

**Conclusion:**

Two novel *CFTR* mutations in exons 17 and 19 are responsible for CF with severe phenotypes in two Iranian families. These two mutations supplement the mutation spectrum of *CFTR* and may contribute to a better understanding of CFTR protein function.

## INTRODUCTION

Cystic fibrosis (CF) (MIM 219700) is a common autosomal recessive disorder that affects many different organs[1,2]. The leading cause of morbidity and mortality is the progressive decline in pulmonary function resulting from airway damage caused by thickened secretions complicated by chronic microbial infection[3]. Moreover, the other clinical symptoms of the CF patients include insufficiency of the exocrine pancreas in about 85% of CF patients, meconium ileus in nearly 15%, diabetes mellitus in 15% and severe liver disease in about 5%. Furthermore, 99% of CF males are infertile because of congenital bilateral absence of the vas deferens[4].

CF is common among Caucasians of Northern European descent, with about 1/2500 affected and a carrier rate of about 1/25[5,6]. However, other ethnic and racial groups are less commonly affected. For example, the prevalence of CF among African-Americans is approximately 1/17,000, which corresponds to a carrier rate of 1/65[7]. Few reports have described the distribution and abundance of cystic fibrosis transmembrane conductance regulator (*CFTR*) gene (MIM 602421) mutations in Iranian patients[8-12].

A study on 37 Iranian CF patients in 2004 detected six mutations, including p.F508del, p.W1282X, p.G542X, p.R117H, p.R347H and p.A120T[11]. Another study on 69 Iranian CF patients identified 37 mutations, of which the p.F508del was the most frequent mutation[12]. In a recent study performed on a northern Iranian population, the p.F508del mutation was also the most frequent[13].

At the molecular level, a defective CFTR protein leads to inadequate transport of chloride ions between the intra- and extra-cellular environments of epithelial cells in affected organs. In pancreatic ducts, the same defect leads to inspissated secretions, which blocks the duct and prevents the transport of pancreatic enzymes into the digestive tract. The biliary tree, vas deferens and sweat ducts are likewise compromised. CF is generated by mutations in the *CFTR* gene. In 1989, in a collaborative study, Kerem and colleagues identified the gene responsible for CF[14] and found that, in the majority of CF patients, the gene was missing three nucleotides, which resulted in the in-frame deletion of a phenylalanine residue at position 508 of the polypeptide chain (ΔF508)[15].

The *CFTR* gene is situated at the location 7q31.2 and contains 27 exons. The CFTR protein output is located in the cell membrane and functions as an ion channel. Mutations in the *CFTR* gene affect the protein function and cause CF symptoms[16-19].

CF is characterized by substantial allelic heterogeneity, with more than 2000 different mutations (Cystic Fibrosis Mutation Database, www.genet.sickkids.on.ca/cftr/) reported within the *CFTR* gene[1]. Approximately 50% of Caucasian CF patients are homozygous for the ΔF508 mutation, which results in complete loss of CFTR function and classic, severe manifestations of the disease. About 40% of CF patients have ΔF508 on one chromosome and another less common mutation on the other chromosome. The remaining ~10% has two rare mutations[20,21].

Previous research in the Iranian population indicated that ΔF508 is the most common mutation in the Iranian population and in other populations, and all known mutations have a high heterogenic frequency in Iran[22]. Therefore, the current study aimed at defining the molecular aspects of CF in Iran.

## MATERIALS AND METHODS

We investigated two previously diagnosed Iranian families with a history of CF, who were referred to the Kariminejad-Najmabadi Pathology and Genetics Center, Tehran, Iran for molecular diagnostic and carrier testing. The first family had a 5-month-old child, who was affected with CF and had passed away; therefore, his parents were referred to this center for carrier detection (Family I). The patient in the other family had CF symptoms with a strong suspicion of CF disease (Family II). The patient in Family I had a clinical diagnosis of CF according to both the clinical presentation and the results of repeated sweat tests (quantitative pilocarpine iontophoresis[23]) and was hence defined as a “sweat test confirmed” CF patient. The patient in Family II was suspected of having atypical CF with equivocal sweat test results and a single CF symptom.

### Genetic analysis

After genetic counseling, a blood sample (10 mL) was collected from each patient, and genomic DNA was extracted[24]. This was followed by PCR and reverse hybridization using the *CF* StripAssay (ViennaLab Diagnostics, Vienna, Austria) to detect the following 35 common mutations: CFTRdel2,3 (21Kb) (c.54-5940_273+10250del); I507del (-ATC) (c.1519_ 1521delATC); F508del (-CTT) (c.1521_1523delCTT); 1717-1G>A (c.1585-1G>A); G542X (c.1624G>T); G551D (c.1652G>A); R553X (c.1657C>T); R560T (c.1679G>C); 2143delT (c.2012delT); 2183AA>G (c.2051_2052delAAinsG); 2184delA (c.2052delA); 2184delA (c.2052delA); 2184insA (c.2052_2053insA); 2789+5G>A (c.2657+5G>A); R1162X (c.3484C>T); 3659delC (c.3528delC); 3905insT (c.3773dupT); W1282X (c.3846G>A); N1303K (c.3909C>G); G85E (c.254G>A); 394delTT (c.262_263delTT); R117H (c.350G>A); Y122X (c.366T>A); 621+1G>T (c.489+ 1G>T); 711+1G>T (c.579+1G>T); 1078delT (c.948delT); R334W (c.1000C>T); R347H (c.1040G> A); R347P (c.1040G>C); A455E (c.1364C>A); 1898+1G>A (c.1766+1G>A); 3120+1G>A (c.2988+ 1G>A); 3272-26A>G (c.3140-26A>G); Y1092X (c.3276C>A); 3849+10KbC>T (c.3718-2477C>T).

The samples were further analyzed by Sanger sequencing for all 27 exons, intronic flanking regions, and 5’ and 3’ UTRs of the *CFTR* gene using the ABI PRISM™ BigDye Terminator Cycle Sequencing kit and the ABI PRISM™ 3130-Avant Genetic Analyzer (Applied Biosystems, Foster City, CA, USA). Alleles were discriminated using CodonCode Aligner software version 6.0.2. The pathogenicity of novel variants was predicted using bioinformatics software such as PolyPhen, Conseq, Sift and MutationTaster. The study was approved by the University of Social Welfare and Rehabilitation Sciences Institutional Ethics Committee for Research Protocols.

## RESULTS

### Family I

A healthy and unrelated couple, a 36-year-old male and a 27-year-old female, both with Persian ancestry, was referred to our center by a gastroenterologist and gynecologist for diagnostic testing. They were referred as a result of CF diagnosis in their first child who died at the age of five months and who was the only affected subject in this family. His first symptom was steatorrhea at 17 days of age, and he presented pulmonary symptoms such as cough. Ultrasound investigations revealed posterior urethral valves and hernia, and so he was diagnosed with CF early after birth.

**Fig. 1:**
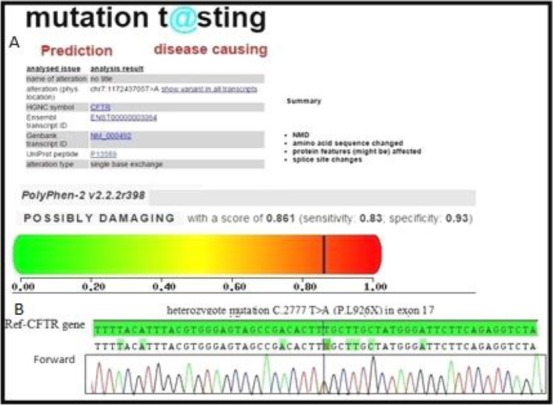
Characterization of a novel nonsense variant identified in CFTR gene. (A) MutationTaster and PolyPhen results for the detected substitution showing the c.2777T>A nonsense mutation might be a disease causing mutation. (B) Confirmation of a heterozygous mutation by direct sequencing; a thymine-to-adenine substitution occurs at nucleotide 2777 in exon 17 (c.2777T>A).

In molecular assessment of father, we found a new heterozygous nonsense mutation in exon 17 of the *CFTR* gene, which changed T to A at position 2777 defined as (c.2777 T>A (p.L926X)). To predict the pathogenicity of this mutation, *in silico* analysis software such as MutationTaster, PolyPhen-2 and Sift was used. MutationTaster showed that the mutation was disease causing and might affect protein features, and the result was confirmed by PolyPhen-2 software (Fig. 1). Furthermore, a heterozygous splice site mutation in the *CFTR* gene, defined as c.1393-1G>A and described previously as a pathogenic mutation, was found in the mother by direct sequencing.

### Family II

The patient, a 32-month-old male, was the first child of non-consanguineous Persian parents, who referred to our center for *CFTR* DNA analysis. This family had only one affected individual with CF symptoms. The molecular finding in this patient showed a compound heterozygous mutation in exon 19 of the *CFTR* gene, defined as one novel missense mutation changing T to A at position 3119 (c.3119T>A), which caused the substitution of leucine to histidine at position 1040 (c.3119T>A (p.L1040H)) and also one previously reported nonsense mutation c.3484C>T (p.R1162X) in exon 22 of *CFTR* gene.

**Fig. 2:**
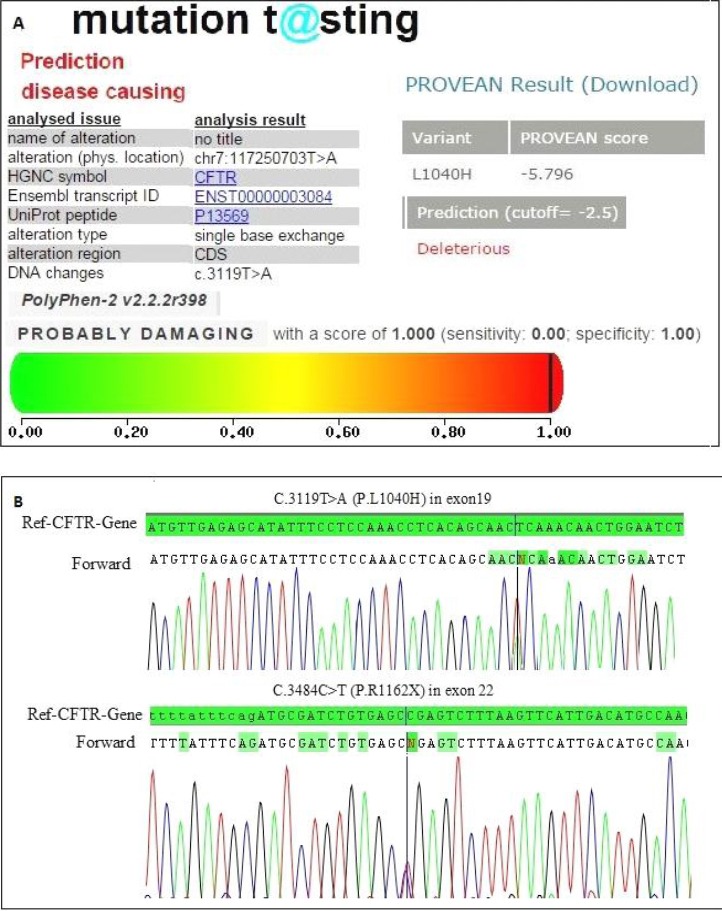
Characterization of a novel missense variant identified in *CFTR* gene. (A) MutationTaster, Sift and PolyPhen results showing c.3119T>A to be a disease causing mutation. (B) Confirmation of a compound heterozygous mutation by direct sequencing; a novel thymine-to-adenine substitution occurs at nucleotide 3119 in exon 19 (c.3119T>A) and a previously reported cytosine-to-thymine substitution at nucleotide 3884 in exon 22 (c.3484C>T).

To confirm the novel mutation c.3119T>A, we used *in silico* analysis tools. The results predicted that this mutation could be a causative mutation (Fig. 2), which may confirm the clinical diagnosis for this patient.

## DISCUSSION

In this study, we report two novel mutations, one nonsense in a healthy adult male (having an infant died at five months) who was referred for carrier detection, and one missense in a 2.5-year-old child with a definite clinical length diagnosis of CF. To date, over 2000 mutations have been identified in the *CFTR* gene; almost all are point mutations or small (1–84 bp) deletions. Mutations in the *CFTR* gene can be categorized based on the disruption in CFTR protein function[25,26]. Mutations in Class I result in premature truncation of nascent CFTR polypeptide and lead to little or no protein expression and cause severe disease in the homozygous form or compound heterozygous form in combination with a Class II mutation. Class II mutations affect the synthesis of CFTR protein. The homozygous form of this mutation can lead to the development of severe disease (F508del)[1]. Class III mutations alter CFTR gating and result in lowered Cl^−^ transport, despite the expression of full protein at the apical plasma membrane of epithelial cells. Class IV mutations can cause reduced Cl permeability. Mutations in Class V induce decreased expression of CFTR protein, and with Class VI mutations, the protein has abnormally short residence time at the apical plasma membrane[27,28]. Individuals who carry Class IV–VI mutations often have milder disease. Although genotype-phenotype correlations in CF are imprecise[29], a CF patient’s clinical phenotype will usually reflect either full loss of or some fractions of CFTR ion transport function if there is residual ion transport function afforded by one of the mutant *CFTR* alleles[30].

In this study, we screened all exons and splicing sites in the *CFTR* gene in two families, and two novel mutations were identified. A substitution of leucine to stop codon at position 926 in the *CFTR* gene (p.L926X) occurred in the transmembrane domain of the *CFTR* gene in the first family. The replacement of leucine to histidine at position 1040 (p.L1040H) of the *CFTR* gene occurred in the topological domain of the CFTR protein in the second family. We also found two previously reported mutations (c.1393-1G>A in Family I and c.3484C>T in Family II). Bioinformatics analysis showed that the two novel mutations were located in the transmembrane domains; these regions play a major role in the regulation of pore function in CFTR protein (CFTR admin database) so these mutations can damage CFTR protein function. Pathogenesis of these variants was evaluated by mutation classification, bioinformatic methods and also normal population study. Considering the clinical presentation and *in silico* software analyses such as dbSNP, Sift, PolyPhen, MutationTaster and our Iranian polymorphism database consisting of 400 normal ethnically adjusted samples (normal population study), the two novel mutations L926X and L1040H might not be polymorphisms, and they are presumably pathogenic mutations (Figs. 1 and 2). Since these two novel mutations are located in the transmembrane domain, this could cause lowered Cl^−^ transport; therefore, these mutations could be considered to be a Class III mutation type.

Three changes have been reported at protein position 926: p.Leu926AlafsX48 (c.2775_2776delTT), p.Leu926CysfsX16 (c.2777delT) and p.Leu926Phe (c.2778G>T), of which two are frameshift mutations caused by thymine deletions. The only reported mutation at amino acid position 1040 is p.Leu1040Phe, which is a G to T substitution at location 3118 (c.3118C>T).

The two previously reported mutations found in this study were c.1393-1G>A and c.3484C>T (p.R1162X) in two families. In Family I, c.1393-1G>A is a splice site variant in intron 10, which is not prevalent in the general population. In a recent study, the c.1393-1G>A has been shown that to be one of the most frequent mutations in CF patients[31]. In Family II, R1162X is a nonsense mutation with substitution of arginine to stop codon at position 1162, and with a relative frequency of 0.3% in the general population. This mutation is among the panel of 10 core mutations that the ACMG CF Carrier Screening Working Group recommended to be screened during routine CF diagnostic testing and carrier screening in the general population[32]. This mutation has also been reported from Shiraz city, Fars Province in the south of Iran[33]. It has been reported that the R1162X transcript is stable and the truncated protein is probably misfolded; therefore, it is likely categorized in Class II. Our finding in Family II carrying two severe or classic mutations (Class II and III type) confirms the definitive and classic phenotype in this patient.

In Iran, complete genetic information is currently lacking to implement solid population-based *CFTR* screening programs that could enable adequate carrier detection of either typical or atypical CF patients or their family members. For national policies of CF prevention, it is acceptable to include only the most frequent mutations present in the population, which allows a 90% detection rate.

In conclusion, two novel *CFTR* mutations in the transmembrane domain and topological domain have been identified in CF families, which may extend the mutation spectrum of CF and contribute to better molecular understanding of the involvement of the *CFTR* gene. Additionally, this knowledge will help in developing new strategies to improve and extend the number of mutations screened for prenatal diagnosis and carrier screening.
